# Analysis of Fish-Eye Lens Camera Self-Calibration

**DOI:** 10.3390/s19051218

**Published:** 2019-03-10

**Authors:** Kang Hyeok Choi, Yongil Kim, Changjae Kim

**Affiliations:** 1 Department of Civil &Environmental Engineering, Seoul National University, 599 Gwanak-ro 1, Gwanak-gu, Seoul 08826, Korea; cwsurgy@naver.com (K.H.C.); yik@snu.ac.kr (Y.K.); 2 Department of Civil and Environmental Engineering, Myongji University, 116 Myongji-ro, Cheoin-gu, Yongin, Gyeonggi-do 17058, Korea

**Keywords:** fish-eye lens camera, perspective projection camera, self-calibration, interior orientation parameters, correlation analysis, test object shapes

## Abstract

The fish-eye lens camera offers the advantage of efficient acquisition of image data through a wide field of view. However, unlike the popular perspective projection camera, a strong distortion effect appears as the periphery of the image is compressed. Such characteristics must be precisely analyzed through camera self-calibration. In this study, we carried out a fish-eye lens camera self-calibration while considering different types of test objects and projection models. Self-calibration was performed using the V-, A-, Plane-, and Room-type test objects. In the fish-eye lens camera, the V-type test object was the most advantageous for ensuring the accuracy of the principal point coordinates and focal length, because the correlations between parameters were relatively low. On the other hand, the other test objects were advantageous for ensuring the accuracy of distortion parameters because of the well-distributed image points. Based on the above analysis, we proposed, an accurate fish-eye lens camera self-calibration method that applies the V-type test object. The RMS-residuals of the proposed method were less than 1 pixel.

## 1. Introduction

The fish-eye lens camera has the advantage of having, relative to a conventional optical camera, a wider viewing angle for recording of color (RGB) information within a wide area, specifically by compressively recording the outer part of the camera image relative to the center part. Due to these advantages of the fish-eye lens camera, it has been used for indoor and outdoor 3D modeling, augmented reality, and Simultaneous Localization And Mapping (SLAM) in the fields of remote sensing, surveying, and robotics. Marković et al. [1] and Caruso et al. [2] used a fish-eye lens camera for SLAM and mobile robot implementation, and Sánchez et al. [3] used a fish-eye lens camera to improve the accuracy of urban navigation. Schöps et al. [4] used a fish-eye lens camera to produce a three-dimensional model for a large area. Gao et al. [5] and Yang et al. [6], meanwhile, developed a driver-assisted sensor system consisting of fish-eye lens cameras.

Although the fish-eye lens camera has a wide Field Of View (FOV), it has the disadvantage of strong distortion in the image. Therefore, self-calibration must be employed to correct the image distortion [7,8]. Self-calibration is the process of determining the Interior Orientation Parameters (IOPs) of the sensor. The IOPs include principal point coordinates ($x_{p}$, $y_{p}$), focal length (f), and distortion parameters.

Fish-eye lens distortion is represented by two components, the projection model and lens distortion. Distortion due to the projection model is the intended distortion in the design and fabrication stages of the fish-eye lens camera, which results in a larger distortion effect from the center of the image to the outer edge. On the other hand, the fish-eye lens camera, also, has lens distortion phenomenon, which is the same as conventional optical cameras [9]. Lens distortion models provide interpretations of radial and decentering distortions, which are not explained in the projection model and have been verified through a variety of studies including Brown [10], Beyer [11], and Fraser [12].

There are four typical projection models of fish-eye lens cameras: equidistant, equisolid-angle, orthogonal, and stereographic projection [13,14,15,16]. In addition to the typical projection model, various projection models such as Fish Eye Transform (FET), Polynomial Fish Eye Transform (PFET) [17,18], FOV [19], and Division model [20,21] has been proposed.

### 1.1. Previous Studies

The fact that each projection model of the fish-eye lens camera can adequately explain the geometric distortion of the fish-eye image has been verified through various self-calibration studies of fish-eye cameras, as follows. Li [22] and Chunyan et al. [23] performed self-calibration of a panoramic camera by applying the equidistant projection model and lens distortion model. Hughes et al. [24] proposed a method to extract the segment information from the image of the equidistant projection fish-eye lens camera to calculate the vanishing point and to remove the distortion effect. Sahin [25] performed a calibration on a mobile phone camera using an equidistant projection model. Schneider et al. [26] fabricated a multi-sensor system consisting of an equisolid-angle projection camera and a lidar and calibrated the involved sensors. Perfetti et al. [27] performed stereoscopic projection fish-eye lens camera calibration and used three-dimensional models of cultural property buildings using the corrected images. Bakstein et al. [28] presented a new projection model combining a stereographic and equisolid-angle projection model and used it to perform a fish-eye lens camera calibration. Xiong et al. [29] used a PFET-lens distortion model to perform self-calibration of a fish-eye lens camera to be used for indoor mobile-robot positioning.

In addition to the verification of each projection model of the fish-eye lens, comparisons between the projection models and the possibility of substituting the projection model also have been explored, as follows. Schneider et al. [30] and Hughes et al. [9] performed self-calibration for various fish-eye lens cameras of different projection models and compared their accuracies. Schneider et al. [30] conducted self-calibration of four representative projection models (equidistant, equisolid-angle, orthogonal, and stereographic projection) and found that when using a model that is not an actual projection model, distortion correction was possible through the adjustment of the lens distortion parameter, but it was confirmed that the principal point coordinates or the focal length, was not calculated accurately. Hughes et al. [9] compared self-calibration accuracy among equidistant, equisolid-angle, orthogonal, stereographic, FET, PFET, FOV, and division projection models. Marcato Junior et al. [8] studied self-calibration when no information about the fish-eye lens camera projection model was provided in advance. The authors considered the perspective projection model and other various fish-eye lens projection models to find the appropriate projection model for their study. They also found that the combination of perspective projection and lens distortion models cannot accurately account for fish-eye lens camera distortion. 

The previous studies have served to demonstrate how accurately each projection model can interpret the geometric distortion of a fish-eye lens camera. However, a number of them found a correlation between IOPs and Exterior Orientation Parameters (EOPs) [8,30,31], which has rarely been analyzed in detail.

The correlation between the camera’s orientation parameters is very important indicator of the calibration accuracy, because it has a very large impact on the self-calibration results. High correlation between the orientation parameters significantly decreases the reliability of the calibration result, and the other way around [32,33].

Correlation between orientation parameters is also observed in the case of a normal lens camera (perspective projection camera). In the perspective projection model camera, a calibration methodology for reducing the correlation between parameters has been proposed in various papers [32,33,34,35,36,37,38,39]. Conventional methods lower the correlation between orientation parameters by implementing a network geometry that is advantageous for self-calibration, specifically by adjusting the relative position and orientation between the calibration target and the camera. Representative conventional methods include: (1) using both of landscape and portrait images taken and (2) photographing the target in oblique directions [32].

On the other hand, the fish-eye lens camera differs from the conventional camera in the projection model equation, the way in which the correlation occurs may also be different, which fact can have a significant impact on the self-calibration accuracy of the fish-eye lens camera. Therefore, a careful analysis of the projection model of the fish-eye lens camera, and the effect of the correlation on the self-calibration of the fish-eye lens camera are needed.

### 1.2. Purpose of Study

The objectives of this study are: (1) to analyze the effect of different image data acquisition method (i.e., change of test object type or camera shooting method) on the correlation between orientation parameters, and (2) to formulate a self-calibration method that can accurately derive the IOPs of the fish-eye lens camera.

The partial derivative of the projection model was performed to determine the interaction effects between the orientation parameters. In this study, it was performed on five projection models, one of which is perspective projection and the other four being representative projection models of the fish-eye lens camera (equidistant, equisolid-angle, orthogonal, and stereographic projection). The derived partial derivatives were used to analyze the causes of different correlations between projection models.

Self-calibration and correlation analysis of camera orientation parameters were performed through simulation. At this stage, one should note that the simulation is carried out using the systematic and stochastic models which have been well-established and verified in the photogrammetric community. The reason for conducting the experiments through simulation is that the simulation test has the following advantages. First, the locations of the camera and test object can be set to exact values in the simulation. In other words, simulation is easy to perfectly control all the involved orientation parameters. Contrarily, in the case of self-calibration using real data, there is a limitation in the accurate setting of the positions of the camera and the test object, because there is an error in the setting of each variable even though it is a sophisticated experiment. Next, simulation can more clearly confirm the accuracy of results. In other words, since the simulation is carried out after setting all the involved variables, the estimated values from self-calibration can be directly compared and analyzed with the preset values. Such comparison is almost impossible in the case of real data. Lastly, simulation can handle different types of test objects, camera shooting positions, and looking angles without any limitation. Eventually, the results from simulation can reduce the time and economic costs of real experiments. 

This paper describes the research contents in the following order. Section 2 describes the mathematical model and partial derivatives of the fish-eye lens camera. Section 3 and Section 4 explain the design of a self-calibration simulation and analyze the experimental results. Section 5 proposes a method for securing the accuracy of fish-eye lens camera self-calibration. Finally, Section 6 draws conclusions on the findings of this research and looks ahead to upcoming work. 

## 2. Analysis of the Camera Mathematical Model

This section, first, introduces the mathematical models of five different camera types. Projection and lens distortion model are addressed. Afterwards, the derivation and analysis of the partial derivative for the projection models of the cameras follow. 

### 2.1. Mathematical Model of Camera

The mathematical model of a camera is divided into a projection model equation and a lens distortion model equation. Typical projection models of cameras are perspective (normal lens camera), equidistant, equisolid-angle, orthogonal, and stereographic projection models (fish-eye lens cameras). Each projection model is represented by camera image coordinates, object points, IOPs, and EOPs. The lens distortion model, which explains the distortion caused by the camera lens, is used in both the perspective projection camera and the fish-eye lens camera. 

#### 2.1.1. Projection Models

**Figure 1:**
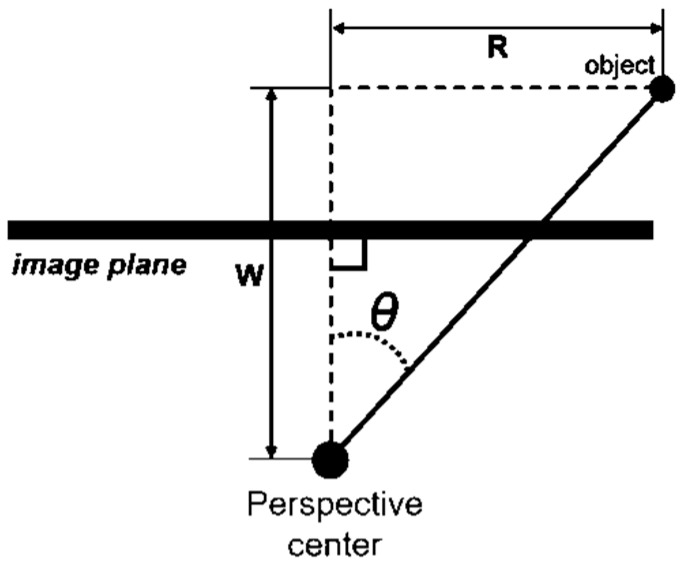
Incident angle (θ) in imaging geometry.

The image coordinates (x, y) of the camera are calculated as shown in Equations (1) to (13) and Figure 1. Where ($x_{p}$, $y_{p}$) are the coordinates of the principal point, r is the distance from the principal point to the image point, f is the focal length, θ is the incident angle of the object point, (X, Y, Z) are the coordinates of the object point, and (X0, Y0, Z0, ω, φ, κ) are EOPs:

(1) $$x=x_{p}-\frac{r}{R}U$$

(2) $$y=y_{p}-\frac{r}{R}V$$

(3) $$R=\sqrt{U^{2}+V^{2}}$$

(4) U=m11(X−X0)+m12(Y−Y0)+m13(Z−Z0)

(5) V=m21(X−X0)+m22(Y−Y0)+m23(Z−Z0)

(6) W=m31(X−X0)+m32(Y−Y0)+m33(Z−Z0)

(7) $$Rotation\;matrix\;M\left(\omega ,\varphi ,\kappa \right)=\left[\begin{array}{ccc} m11 & m12 & m13\\ m21 & m22 & m23\\ m31 & m32 & m33 \end{array}\right]$$

(8) If Perspective Projection (Normal lens) r=ftanθ

(9) Else if Equidistant Projection (Fish-eye lens) r=fθ

(10) $$\mathrm{Else}\;\mathrm{if}\;\mathrm{Equisolid}-\mathrm{angle}\;\mathrm{Projection}\;(\mathrm{Fish}-\mathrm{eye}\;\mathrm{lens})\;r=f2\sin\frac{\theta }{2}$$

(11) Else if Orthogonal Projection (Fish-eye lens) r=fsinθ

(12) $$\mathrm{Else}\;\mathrm{if}\;\mathrm{Stereographic}\;\mathrm{Projection}\;(\mathrm{Fish}-\mathrm{eye}\;\mathrm{lens})\;r=f2\tan\frac{\theta }{2}$$

(13) $$\theta =\arctan\left(\frac{R}{W}\right)$$

#### 2.1.2. Lens Distortion Model

The mathematical model of the lens distortion is given by Equations (14) to (18), where Δx and Δy are distortions of the image coordinates x and y. (K_1_, K_2_, K_3_) are the radial lens distortion parameters; (P_1_, P_2_) are the decentering distortion parameters; and (A_1_, A_2_) are the terms for affinity and shear. r in Equations (8) to (12) can be rewritten as the distance between the image coordinates (x, y) and the principal point coordinates ($x_{p}$, $y_{p}$) as described in Equation (18).

The mathematical model of a camera is expressed using the projection model Equations (1) and (2) along with the distortion model Equations (14) and (15). Equations (19) and (20) are the final mathematical models of the camera. The image coordinates x and y of the camera are represented by object point coordinates, IOPs, EOPs, and lens distortions:

(14) $$\Delta x=\bar{x}\left(K_{1}r^{2}+K_{2}r^{4}+K_{3}r^{6}\right)+P_{1}\left(r^{2}+2{\bar{x}}^{2}\right)+2P_{2}\bar{x}\bar{y}+A_{1}\bar{x}+A_{2}\bar{y}$$

(15) $$\Delta y=\bar{y}\left(K_{1}r^{2}+K_{2}r^{4}+K_{3}r^{6}\right)+2P_{1}\bar{x}\bar{y}+P_{2}\left(r^{2}+2{\bar{y}}^{2}\right)$$

(16) $$\bar{x}=x-x_{p}$$

(17) $$\bar{y}=y-y_{p}$$

(18) $$r=\sqrt{{\bar{x}}^{2}+{\bar{y}}^{2}}$$

(19) $$x=x_{p}-\frac{r}{R}U+\Delta x$$

(20) $$y=y_{p}-\frac{r}{R}V+\Delta y$$

### 2.2. Projection Model Partial Derivative

In this study, partial derivatives were derived using the camera projection models for the mathematical analysis of the tendency of the correlation change according to the test object and imaging method. Partial derivatives provide information on the effect of one variable on the increase/decrease of the other variable, so that the partial differential value is highly correlated with the correlation between the two variables.

**Figure 2:**
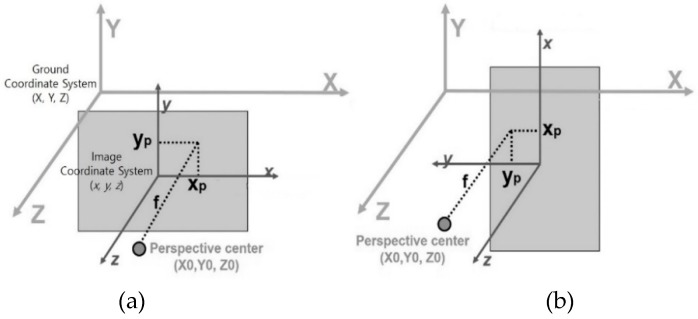
Ground and image coordinate system when (**a**) κ = 0°, (**b**) κ = 90°.

In this study, we determined the combination of the orientation parameters needed to perform the partial differentiation based on the ground and image coordinates as shown in Figure 2. Figure 2a, b show the ground and image coordinate system when EOP κ at 0° and 90°, respectively, where x, y, and z are image coordinates, and X, Y, and Z are ground coordinates.

In Figure 2a, $x_{p}$, $y_{p}$, and f change in the same direction as X0, Y0, and Z0, respectively. Also, in Figure 2b, $x_{p}$, $y_{p}$, and f change in the same direction as Y0, X0, and Z0, respectively. Therefore, five partial derivatives ($\frac{\partial x_{P}}{\partial X0}$, $\frac{\partial x_{P}}{\partial Y0}$, $\frac{\partial y_{P}}{\partial X0}$, $\frac{\partial y_{P}}{\partial Y0}$ and $\frac{\partial f}{\partial Z0}$) were considered in this study. 

Equations (21) to (23) are derived from Equations (19) and (20). Afterwards, the abovementioned five partial derivatives are derived from Equations (21) to (23) and shown in Table 1. Where $r^{\prime }$ is equal to r (seen in Equations (8) to (12)) divided by focal length (f):

(21) $$x_{p}=x+\frac{r}{R}U-\Delta x$$

(22) $$y_{p}=y+\frac{r}{R}V-\Delta y$$

(23) $$f=\frac{R}{r^{\prime }U}\left(x_{p}+\Delta x-x\right)\;\mathrm{or}\;f=\frac{R}{r^{\prime }V}\left(y_{p}+\Delta y-y\right)\;\left(r^{\prime }=\frac{r}{f}\right)$$

More specifically, Equations (24) to (31) were obtained by partial derivation of Equations (21) and (22) with X0 and Y0, and Equation (32) was the result obtained by partial derivation of Equation (23) with Z0. Equations (24) to (27) are partial derivatives when ω, φ, and κ, which are the rotational components of the EOPs, are all set to 0 °, and in Equations (28) to (31), ω and φ are set to 0° and κ is set to 90°. Where $\bar{X}$ and $\bar{Y}$ are respectively the difference between object point coordinates (X and Y) and EOP (X0 and Y0). Hence, $\bar{X}=\left(X-X0\right)$ and $\bar{Y}=\left(Y-Y0\right)$. Equations (24) to (32) show two important characteristics that suggest that the correlation between the orientation parameters of the perspective projection camera and the fish-eye lens camera may be different.

The details of each characteristic are as follows. First, Equations (24) to (32) all have r and $\frac{\partial r}{\partial \theta }$ in common. r varies depending on the projection model, which means that the effect of one orientation parameter change on the other orientation parameter differs depending on the projection model. Next, in the perspective projection camera, Equations (25), (26), (28), and (31) can be all zeros, and a partial value of 0 means that there is no correlation between the orientation parameters. In these cases, when κ is 0°, there is no correlation between $x_{p}$ and Y0 or between $y_{p}$ and X0, and when κ is 90°, there is no correlation between $x_{p}$ and X0 or between $y_{p}$ and Y0.

## 3. Self-Calibration Design

**Figure 3:**
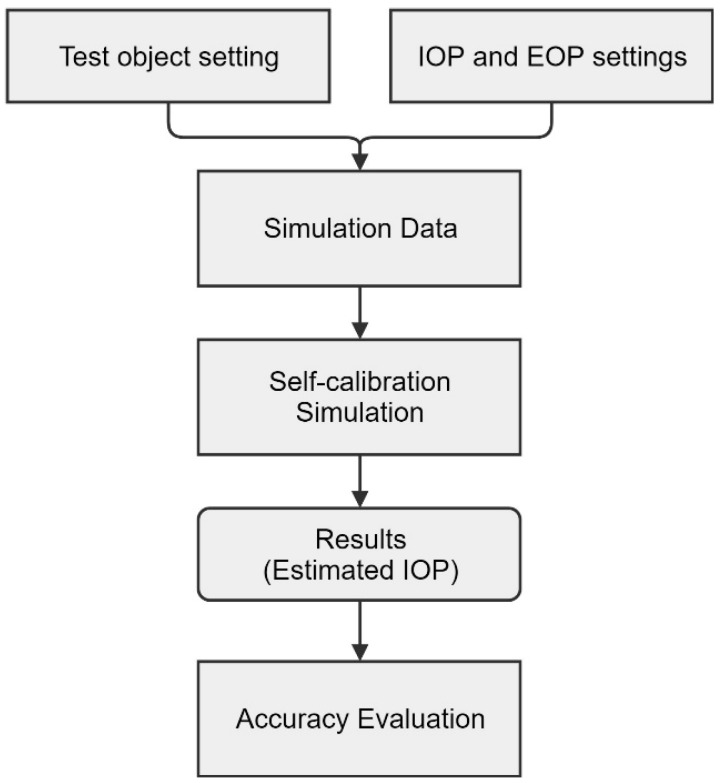
Flow chart of self-calibration simulation.

The flow of the self-calibration simulation is shown in Figure 3. First, the test object type and orientation parameters (IOPs and EOPs) were set up, and the simulation datasets were created using the parameters. Next, the self-calibration was performed using the prepared data. Finally, the estimated IOPs were evaluated for accuracy through each predefined orientation parameters.

**Figure 4:**
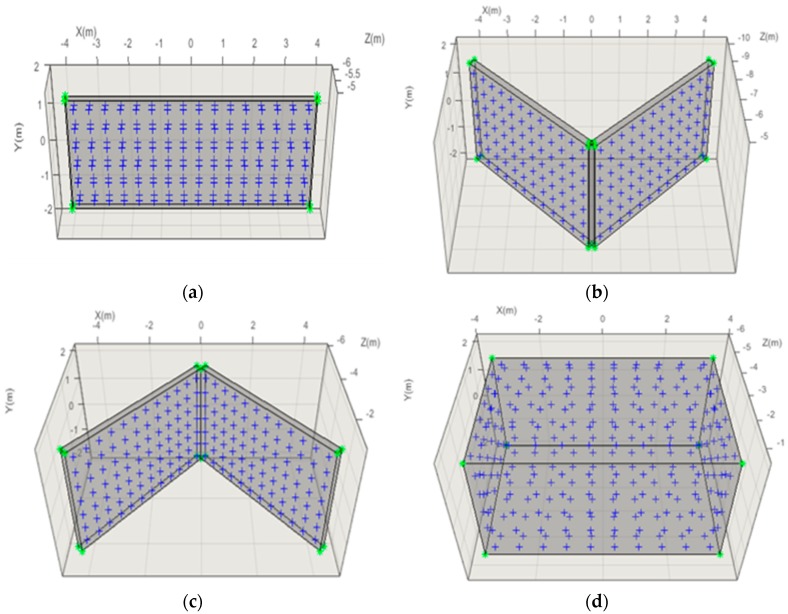
Test object shapes: (**a**) Plane-, (**b**) V-, (**c**) A-, and (**d**) Room-type.

The four different types of test objects: Plane, V, A, and Room, were used in the self-calibration. The dimensions and characteristics of the test objects are shown in Table 2 and Figure 4. Figure 4a–c show a frontal view of the Plane, V, and A type test objects, respectively, and Figure 4d shows the Room-type test object.

The IOPs and camera specification for the self-calibration simulation are set as shown in Table 3. Each value in Table 3 was set based on a sunnex DSL315 fish-eye lens and a Chameleon3 5.0 MP camera body. 

**Figure 5:**
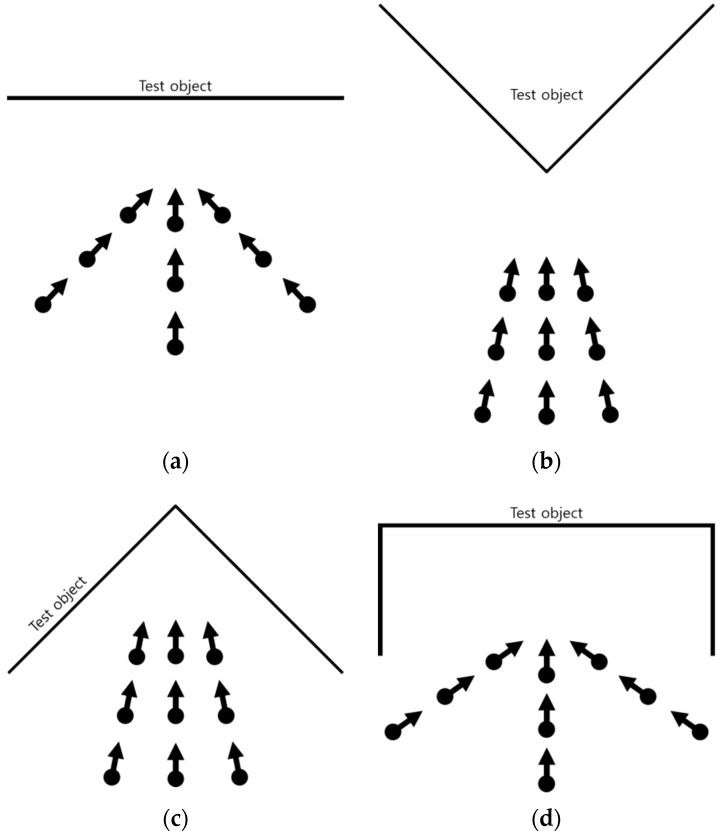
Configuration of image acquisition according to different test object types: (**a**) Plane-, (**b**) V-, (**c**) A-, and (**d**) Room-type.

Table 4 shows the image setting configuration for self-calibration simulation designed in this research: (1) Twenty different cases from four different test object types and five projection models were considered for the self-calibration simulation. (2) Eight self-calibration runs (from four set-A types and four set-B types) for each case were carried out. Hence, one hundred and sixty runs were, totally, implemented in this research. (3) Set-A type includes six images taken at κ = 0° and three images taken at κ = 90°. Also, set-B type includes six images taken at κ = 0° and other six images taken at κ = 90°. Figure 5 shows configuration of image acquisition according to different test object types. Image shooting positions and looking angles were determined to produce convergent images and to view planes as many as possible. Also, both of landscape (κ = 0°) and portrait (κ = 90°) images were taken at the same position. Afterwards, different image-sets were made up of the images taken from different camera shooting positions and looking angles.

## 4. Analysis of Self-Calibration

The accuracy of self-calibration was evaluated for each of the four test object types and five projection models. Accuracy estimates for each case were made through the average of eight results performed using eight image sets. Self-calibration was analyzed through: (i) stability of self-calibration, (ii) correlation between orientation parameters, (iii) accuracy of principal point coordinates and focal length, and (iv) accuracy of distortion parameters. 

### 4.1. Stability of Self-Calibration

Stability of self-calibration was evaluated in three stages: Stable, Unstable, and Divergent, based on whether or not orientation parameters can be solved during the calibration process. The correlation between the orientation parameters was very high, and ‘divergent’ was evaluated when most of the orientation parameters were not solved. Next, when the response was very sensitive to EOPs and divergence occurring according to the combination of images, or when the local optimum problem appeared, it was evaluated as ‘unstable’. Finally, when the calibration was completed without the divergence of the orientation parameters or the local optimal solution, it was evaluated as ‘stable’.

Table 5 shows the stability evaluation results, and it can be seen that the stability of the fish-eye lens camera varies greatly according to the type of test object. In perspective projection, stable self-calibration is performed irrespective of the type of test object, but the fish-eye lens camera cannot have solutions from the A-type and Room-type test object. Next, the Plane-type test object was found to be strongly influenced by EOPs in the calibration of the fish-eye lens camera. In other words, the divergence of the orientation parameters appeared depending on the photographing position, the local optimal solution phenomenon appeared, and the result was unstable. 

### 4.2. Correlation Analysis in Self-Calibration

Table 6, Table 7, Table 8 and Table 9 show the correlation between the orientation parameters in the self-calibration results according to different test object types. The correlation between the orientation parameters was derived after completion (convergence) of the self-calibration. On the other hand, the correlation coefficients came from the first iteration of the self-calibration procedure when it did not provide convergent solutions.

For the perspective projection, at all test object types, the correlation was generally lower than the correlation of the fish-eye lens camera. The maximum correlation value of the perspective projection was shown at f-Z0 when the plane-type test object was used (Table 6), but it was relatively lower than the f-Z0 correlation values of fish-eye projection models. 

For the fish-eye lens projection models, the f-Z0 correlation for each test object showed a large difference, and this tendency explains why the stability evaluation was as shown in Table 5. In the case of divergence in the evaluation of stability (fish-eye self-calibration using the A- and Room-type test objects), the correlation between f and Z0 showed a maximum value of 0.99 (Table 8 and Table 9). In the Plane-type test object, which showed unstable results, the f-Z0 correlation was 0.96~0.97 (Table 6). For the V-type test object, which was evaluated as stable, the correlation was as high as 0.45 (Table 7). That is, the lower the correlation of f-Z0, the more stable the self-calibration.

### 4.3. Accuracy of Principal Point Coordinates and Focal Length

The RMSE of estimated principal point coordinates and focal length were calculated using results of self-calibration which were performed with 8 image-sets for each case. The errors in the estimated principal point coordinates and focal length are shown in Table 10; Table 11, respectively. The cases evaluated as divergence in the stability evaluation were excluded from the evaluation because the orientation parameters diverged and the calibration was not completed normally. The principal point coordinates and focal length showed the largest RMS values of 0.63 and 1.67 pixels, respectively. In other words, both the principal point coordinates and the focal length were estimated with high accuracy.

### 4.4. Accuracy of Distortion Parameters

**Figure 6:**
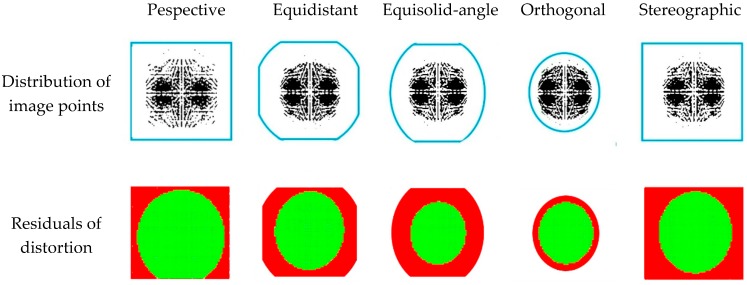
Image point distribution and residuals of distortion.

The accuracy of the distortion parameters was determined by the difference between true distortion at all pixels and estimated distortion (calculated using the estimated distortion parameters). Table 12 shows the RMS-residuals of lens distortion, which indicates that the distortion parameters have the lowest accuracy in the V-type test object. Table 13 and Figure 6 show the reasons for this.

Table 13 shows the ratio of the image points of all images used in self-calibration to the total area of the image. It indicates that the lower the image-coverage ratio of image points, the larger the distortion parameter error. Therefore, the V-type test object has a relatively low coverage ratio, which was interpreted to be lower than those of the other test objects. The A- and Room-type test objects show higher image coverage than other test objects, but distortion parameters are not calculated as orientation parameters diverge. 

Figure 6 shows an example of image point distribution and residuals of distortion for each projection model in the V-type test object. In the figure, ‘Distribution of image points’ shows the distribution of all image points of 12 images (in one image-set) at the same time. The inside of the blue solid line is the region in which the image is recorded. In the row of ‘Residuals of distortion’, green pixels represent residuals of distortion less than 0.5 pixels. And, red pixels indicate the opposite case. This figure shows that the residuals of distortion are relatively high in the region where no image points are distributed.

**Figure 7:**
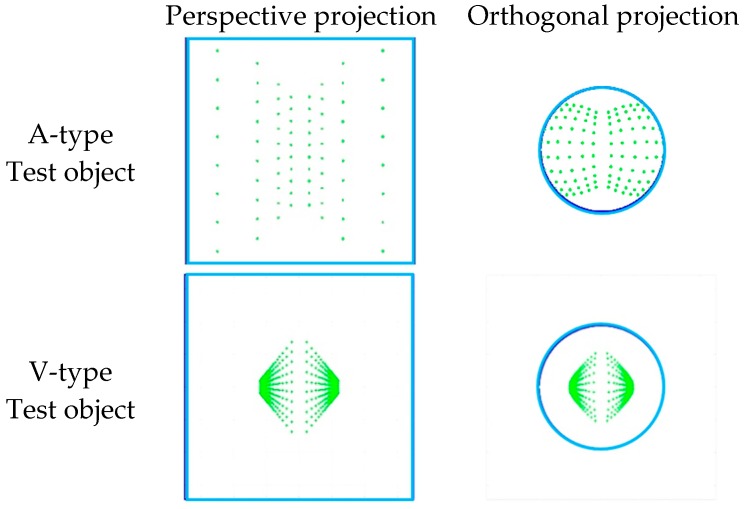
Distribution of image points according to different test object type (A and V).

The reason why the image coverage of the image points in the V-type test object is low is illustrated in Figure 7. It shows images taken from the front of the A- and V-type tests, which were formed of two 20m × 5m Planes. (a) and (c) are perspective projection images, and (b) and (d) are orthogonal projection images. In the case of the A-type test object, the image points are well distributed throughout the image, but the V-type test object shows that the image points are distributed only in the center of the image. As a special case, the V-type test object can improve the distribution of image points when the image is taken closely orthogonal to one side of the test object. But, in that case, a similar geometric relationship is obtained when the image is taken orthogonally to the Plane test object, and the correlation between the parameters increases greatly. 

Figure 7 provides additional information that should be considered for real self-calibration. In case of V-type test object, the neighboring image points are very close to each other at the outer part of the image. When such image points appear, it is very difficult to obtain their image coordinates in a real calibration situation, which fact can negatively affect self-calibration results.

### 4.5. Comprehensive Analysis of Self-Calibration

Table 14 shows the RMS-residuals of the IOPs (principal point coordinates, focal length, and distortion parameters). In the case of the V-type test object, the correlation between the orientation parameters was lower than for the other test object types, but the RMS-residuals of the IOPs was high, because the distribution of image points was not suitable for accurate calculation of the distortion parameters. On the other hand, in the case of the fish-eye lens calibration using the A- and Room-type test objects, divergence appeared due to the high correlation between the orientation parameters. 

The analysis of self-calibration according to the test object and projection models of 4.1~4.5 is summarized as follows.

- For the perspective projection model, the type of test object does not significantly affect the self-calibration accuracy. On the other hand, the fish-eye lens projection models are more influenced by the test object type than the perspective projection model.
- The V-type test object has the highest effectiveness in resolving the correlation between the orientation parameters of the fish-eye lens camera. On the other hand, the A- or Room-type test object is relatively inadequate to solve the correlation, especially, for f-Z0.
- For obtainment of the accuracy of the distortion parameters, the V-type test object is inadequate compared with the other test objects. This is because, in the case of the V-type test object, it is difficult to acquire images with evenly distributed image points.
- The V-type test object has the lowest coverage ratio of image points; on the other hand, the Plane-, A-, and Room-type test objects have relative high ones.

## 5. Proposed Fish-Eye Lens Camera Self-Calibration Method

In this paper, based on the analysis in Section 4, for proposing an accurate fish-eye lens camera self-calibration method, additional experiments were performed for the four different fish-eye lens projection cameras. The simulation was carried out using V-type test object and three image-sets. The accurate fish-eye lens camera self-calibration method was proposed based on comparative analysis of the accuracy of IOPs and the correlation between parameters.

**Figure 8:**
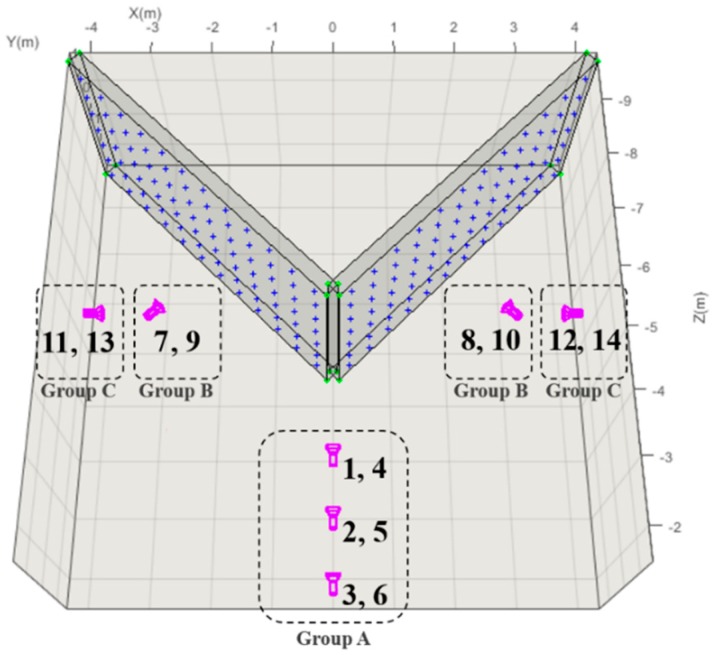
Configuration of image acquisition for additional experiments.

The additional experiments were carried out as follows. Firstly, the image-sets included both of landscape (κ = 0°) and portrait (κ = 90°) images for lowering the correlation of $x_{p}$-X0 and $y_{p}$-Y0. Secondly, the V-type test object was used for reducing the correlation of f-Z0. Lastly, the images viewing two planes of V-type test object, and other ones viewing one plane of its planes, were utilized for estimating distortion parameters accurately. Figure 8 shows the configuration of image acquisition for additional experiments. Table 15, also, explains the image groups shown in Figure 8. The images in Group A (location # 1 to 6) are used for resolving the correlation between parameters. The ones in Group B (location # 7 to 10) and C (location # 11 to 14) are used for estimating accurate distortion parameters. In Group B and C, the images viewing one plane of V-type test object were used to ensure adequate image coverage; this worked similar to the case of the Plane-type test object since only one plane was shown in the images. The images in Group B and C were taken orthogonally and obliquely to the plane, respectively. 

The image-sets utilized for the self-calibration are determined from the combination of A, B and C-image group as seen in Table 16. All the image-sets, commonly, includes Group A because it reduces the correlation between IOPs and EOPs (especially, $x_{p}$-X0, $y_{p}$-Y0, and f-Z0). Image-set 1 and 2 includes Group B and C, respectively. Image-set 3 includes Group A, B, and C; hence, all image groups. The experiments using these three image-sets will be compared and be analyzed to figure out which image-set more effective in accurate self-calibration.

Table 17, Table 18 and Table 19 show the correlation results derived from the self-calibration using different image-sets. The correlation between IOPs and EOPs was low regardless of the image-set. This was because all the image-sets included Group A’s images. Relatively high correlation values (i.e., 0.70–0.72) came from f-Z0 of image-set 1 for all projection models. On the other hand, Image-sets 2 and 3 show low correlation values. Group B’s images in image-set 1, which were taken orthogonally to the plane, deteriorated the decoupling of correlation of f-Z0. Contrarily, Group C’s images in image-sets 2 and 3, which were taken obliquely to the plane, contributed to the decoupling of correlation of f-Z0.

Table 20 and Table 21 show the absolute errors of the estimated principal point coordinates and focal length, respectively. The accuracies of the principal point coordinates were less than 1 pixel in all the cases as seen in Table 20. In the case of focal length, image-set 1 showed mostly higher errors than other image-set cases (Table 21).

**Figure 9:**
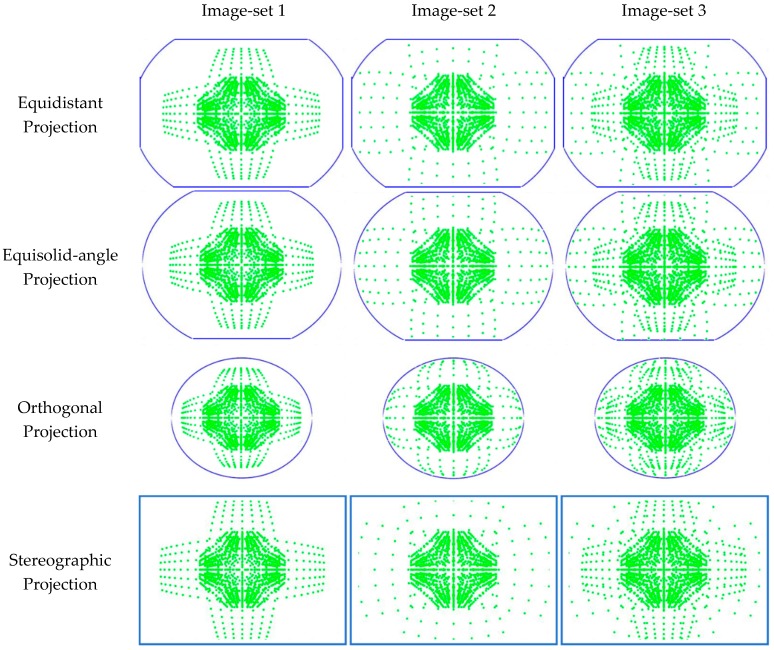
Distribution of image points.

Table 22 shows the RMS-residual of lens distortion. The RMS values of image-set 1 were higher than other image-set cases regardless of the projection models. The worst case came from equidistant model and was 7.48 pixels. In cases of image-sets 2 and 3, the RMS values were significantly improved compared to the case of image-set 1. Table 23 and Figure 9 show the coverage ratio and the distribution of all image points (in one image-set), respectively. As seen in the table and figure, the coverage ratio of image-set 1 was lower than those of image-sets 2 and 3. This was the reason that the residuals of lens distortion were relatively high in the case of image-set 1. 

Table 24 shows the RMS-residuals of the IOPs (principal point coordinates, focal length, and distortion parameters). As seen in the table, image-sets 2 and 3 showed good results with less than 0.85 pixels in all projection models. Image-set 3 (using 14 images) provided slightly better results than image-set 2 (using 10 images). 

In this paper, based on the analysis of additional self-calibration experiments, we proposed the way of deriving accurate fish-eye lens camera self-calibration results as follows:

- Images viewing V-type test object frontally, are used in self-calibration. This leads to reducing correlation between parameters (especially, f-Z0).
- Both of landscape (κ = 0°) and portrait (κ = 90°) images are used in self-calibration. This leads to reducing correlation between parameters (especially, $x_{p}$-X0 and $y_{p}$-Y0).
- Images viewing one plane of V-type test object are used in self-calibration. This ensures adequate image coverage to accommodate lens distortion; hence, it increases accuracies of lens distortion parameters. This effect increases more when dealing with obliques images than orthogonal ones.
- Such images abovementioned can be included in the self-calibration procedure at the same time; and they derive reliable and accurate IOPs.

## 6. Conclusions

This study was conducted in three steps: (i) mathematical analysis of fish-eye lens projection and perspective projection model, (ii) self-calibration analysis of fish-eye lens camera and perspective projection camera in each test-bed type, and (iii) proposed fish-eye lens camera self-calibration method based on analysis of (ii) and additional experiments.

In ‘mathematical analysis for projection model’, the mathematical basis that each projection model shows a different correlation tendency was confirmed based on the partial derivatives of the fish-eye lens projection model. In ‘self-calibration accuracy analysis step’, the V-type test-bed was advantageous in resolving the correlation but was ineffective in the analysis of camera lens distortion. On the other hand, in the case of the A- and Room-type test-beds, because of the very high correlation between focal length and Z0, self-calibration has a high probability of divergence. 

In ‘proposed fish-eye lens camera self-calibration method’, we proposed the fish-eye lens camera self-calibration method to derive high-accuracy IOPs. The proposed method was conducting self-calibration by using the V-type test object as V- and Plane-type. In other words, the proposed method used the images viewing the V-type test object frontally and one plane of the test object in the self-calibration adjustment. Hence, the method adopted the advantages of V- and Plane-type test object at the same time. The fish-eye lens camera self-calibration performed by the proposed method showed the RMS-residuals of less than 1 pixel.

This study will contribute to the self-calibration of the optical camera in the following ways. The results of this study are significant in that they can be a theoretical and empirical basis for a test-bed design that can improve the accuracy of self-calibration of the fish-eye lens camera. Next, we can derive higher-accuracy IOPs through the proposed self-calibration method.

## Figures and Tables

**Table 1:** Partial derivatives considered.

	ω = φ = κ = 0°		ω = φ = 0°, κ = 90°	
$\frac{\partial x_{p}}{\partial X0}$	$=\frac{1}{R^{3}}(r{\bar{X}}^{2}-{\mathrm{rR}}^{2}-\frac{\partial r}{\partial \theta }\cos\theta \sin\theta {\bar{X}}^{2})$	(24)	$=\frac{\bar{X}\bar{Y}}{R^{3}}(r-\frac{\partial r}{\partial \theta }\cos\theta \sin\theta )$ (Perspective projection = 0)	(28)
$\frac{\partial x_{p}}{\partial Y0}$	$=\frac{\bar{X}\bar{Y}}{R^{3}}(r-\frac{\partial r}{\partial \theta }\cos\theta \sin\theta )$ (Perspective projection = 0)	(25)	$=\frac{1}{R^{3}}({r\bar{Y}}^{2}-{\mathrm{rR}}^{2}-\frac{\partial r}{\partial \theta }\cos\theta \sin\theta {\bar{Y}}^{2})$	(29)
$\frac{\partial y_{p}}{\partial X0}$	$=\frac{\bar{X}\bar{Y}}{R^{3}}(r-\frac{\partial r}{\partial \theta }\cos\theta \sin\theta )$ (Perspective projection = 0)	(26)	$=-\frac{1}{R^{3}}({r\bar{X}}^{2}-{\mathrm{rR}}^{2}-\frac{\partial r}{\partial \theta }\cos\theta \sin\theta X^{2})$	(30)
$\frac{\partial y_{p}}{\partial Y0}$	$=\frac{1}{R^{3}}({r\bar{Y}}^{2}-{\mathrm{rR}}^{2}-\frac{\partial r}{\partial \theta }\cos\theta \sin\theta {\bar{Y}}^{2})$	(27)	$=\frac{\bar{X}\bar{Y}}{R^{3}}(r-\frac{\partial r}{\partial \theta }\cos\theta \sin\theta )$ (Perspective projection = 0)	(31)
$\frac{\partial f}{\partial Z0}=-\frac{f}{Z-Z0}\left(\frac{\frac{dr}{d\theta }\cos\theta \sin\theta }{r}\right)$				(32)

**Table 2:** Dimensions of the test objects considered.

Test Object Type	Dimension
Plane	8 × 3.5 (width (m) × height (m))
V	two 6 × 3.5-sized planes (width (m) × height (m))
A	two 6 × 3.5-sized planes (width (m) × height (m))
Room	7 × 5 × 3.5 (length (m) × width (m) × height (m))

**Table 3:** Specification of camera in self-calibration simulation.

**f (mm)**	$x_{p}$ **(mm)**	$y_{p}$ **(mm)**	**Distortion Parameters**						
			**K_1_**	**K_2_**	**K_3_**	**P_1_**	**P_2_**	**A_1_**	**A_2_**
2.9	0.004	0.002	1^−5^	1^−7^	3^−9^	−1^−5^	−2^−7^	1^−5^	2^−7^
**Pixel size (mm)**			**Image Size (pixel)**				**Random Noise (1σ) (pixel)**		
			**x**		**y**				
0.00345			2448		2048		0.5		

**Table 4:** Image setting configuration for self-calibration simulation.

**Number of Test Object Types**	**Number of Projection Models**	**Total Number of Cases**	**Number of Self-Calibration Runs for Each Case**
4^(a)^	5^(b)^	20 cases $\left(=4^{\left(a\right)}\times 5^{\left(b\right)}\right)$	Eight runs from four set-A types and four set-B types
**Image-Set Types**	**Number of Images**		
	$\kappa =0^{{}^{\circ}}$	$\kappa =0^{{}^{\circ}}$	**Total**
set-A type	6	3	9 (= 6 + 3)
set-B type	6	6	12 (= 6 + 6)

**Table 5:** Self-calibration stability.

Projection Model	Test Object Type			
	Plane Type	V Type	A Type	Room Type
Perspective	Stable	Stable	Stable	Stable
Equidistant	Unstable	Stable	Divergent	Divergent
Equisolid-angle	Unstable	Stable	Divergent	Divergent
Orthogonal	Unstable	Stable	Divergent	Divergent
Stereographic	Unstable	Stable	Divergent	Divergent

**Table 6:** Correlation between the orientation parameters (Plane-type test object).

Projection Model	IOP	EOP					
		X0	Y0	Z0	ω	φ	κ
Perspective	$x_{p}$	0.64	0.13	0.10	0.12	0.52	0.04
	$y_{p}$	0.14	0.41	0.17	0.23	0.11	0.02
	f	0.10	0.33	0.93	0.27	0.01	0.01
Equidistant	$x_{p}$	0.58	0.12	0.10	0.11	0.18	0.01
	$y_{p}$	0.14	0.48	0.14	0.13	0.01	0.02
	f	0.11	0.18	0.97	0.13	0.01	0.01
Equisolid-angle	$x_{p}$	0.49	0.12	0.10	0.10	0.16	0.01
	$y_{p}$	0.14	0.33	0.19	0.19	0.03	0.02
	f	0.12	0.18	0.96	0.13	0.02	0.01
Orthogonal	$x_{p}$	0.51	0.12	0.10	0.10	0.35	0.02
	$y_{p}$	0.13	0.29	0.05	0.17	0.01	0.04
	f	0.10	0.31	0.96	0.20	0.01	0.01
Stereographic	$x_{p}$	0.54	0.13	0.10	0.11	0.51	0.02
	$y_{p}$	0.14	0.37	0.16	0.16	0.01	0.03
	f	0.11	0.31	0.97	0.24	0.02	0.01

**Table 7:** Correlation between the orientation parameters (V-type test object).

Projection Model	IOP	EOP					
		X0	Y0	Z0	ω	φ	κ
Perspective	$x_{p}$	0.28	0.12	0.16	0.12	0.66	0.09
	$y_{p}$	0.13	0.21	0.05	0.55	0.12	0.10
	f	0.17	0.12	0.49	0.11	0.03	0.01
Equidistant	$x_{p}$	0.07	0.02	0.04	0.13	0.58	0.09
	$y_{p}$	0.02	0.13	0.03	0.47	0.13	0.10
	f	0.07	0.04	0.43	0.01	0.03	0.01
Equisolid-angle	$x_{p}$	0.07	0.02	0.04	0.13	0.55	0.09
	$y_{p}$	0.02	0.13	0.02	0.46	0.12	0.09
	f	0.07	0.04	0.44	0.01	0.02	0.01
Orthogonal	$x_{p}$	0.07	0.02	0.04	0.13	0.55	0.09
	$y_{p}$	0.02	0.14	0.02	0.56	0.12	0.09
	f	0.07	0.04	0.45	0.01	0.02	0.01
Stereographic	$x_{p}$	0.08	0.02	0.05	0.13	0.58	0.09
	$y_{p}$	0.03	0.12	0.03	0.57	0.13	0.10
	f	0.07	0.03	0.43	0.01	0.03	0.00

**Table 8:** Correlation between the orientation parameters (A-type test object).

Projection Model	IOP	EOP					
		X0	Y0	Z0	ω	φ	κ
Perspective	$x_{p}$	0.29	0.10	0.13	0.11	0.55	0.02
	$y_{p}$	0.06	0.35	0.08	0.51	0.08	0.26
	f	0.14	0.06	0.51	0.02	0.06	0.01
Equidistant	$x_{p}$	0.25	0.15	0.14	0.19	0.49	0.16
	$y_{p}$	0.11	0.30	0.16	0.32	0.20	0.21
	f	0.16	0.17	0.98	0.14	0.07	0.02
Equisolid-angle	$x_{p}$	0.31	0.20	0.10	0.27	0.43	0.25
	$y_{p}$	0.16	0.32	0.16	0.34	0.16	0.21
	f	0.16	0.16	0.99	0.15	0.07	0.03
Orthogonal	$x_{p}$	0.25	0.23	0.16	0.33	0.30	0.34
	$y_{p}$	0.12	0.27	0.11	0.32	0.13	0.19
	f	0.16	0.10	0.98	0.14	0.07	0.05
Stereographic	$x_{p}$	0.30	0.13	0.19	0.13	0.31	0.15
	$y_{p}$	0.19	0.29	0.18	0.27	0.09	0.11
	f	0.17	0.18	0.99	0.17	0.15	0.03

The correlation coefficients of the fish-eye lens projection came from the first iteration.

**Table 9:** Correlation between the orientation parameters (Room-type test object).

Projection Model	IOP	EOP					
		X0	Y0	Z0	ω	φ	κ
Perspective	$x_{p}$	0.28	0.16	0.11	0.18	0.55	0.04
	$y_{p}$	0.06	0.27	0.08	0.30	0.10	0.04
	f	0.11	0.13	0.33	0.11	0.11	0.01
Equidistant	$x_{p}$	0.28	0.12	0.10	0.19	0.42	0.12
	$y_{p}$	0.11	0.29	0.11	0.28	0.18	0.07
	f	0.11	0.12	0.98	0.11	0.12	0.01
Equisolid-angle	$x_{p}$	0.27	0.11	0.13	0.16	0.45	0.19
	$y_{p}$	0.16	0.26	0.15	0.26	0.12	0.11
	f	0.14	0.13	0.99	0.15	0.08	0.16
Orthogonal	$x_{p}$	0.20	0.11	0.15	0.15	0.72	0.09
	$y_{p}$	0.07	0.23	0.13	0.52	0.15	0.30
	f	0.09	0.06	0.98	0.14	0.14	0.09
Stereographic	$x_{p}$	0.24	0.11	0.13	0.06	0.45	0.09
	$y_{p}$	0.06	0.25	0.05	0.26	0.12	0.11
	f	0.14	0.13	0.98	0.15	0.08	0.16

The correlation coefficients of the fish-eye lens projection came from the first iteration.

**Table 10:** RMSE of principal point coordinates (pixel).

Projection Model	Test Object Type			
	Plane	V	A	Room
Perspective	0.38	0.27	0.17	0.20
Equidistant	0.27	0.44	N/A	N/A
Equisolid-angle	0.23	0.63	N/A	N/A
Orthogonal	0.24	0.19	N/A	N/A
Stereographic	0.26	0.29	N/A	N/A

**Table 11:** RMSE of focal length (pixel).

Projection Model	Test Object Type			
	Plane	V	A	Room
Perspective	0.39	0.15	0.05	0.08
Equidistant	1.17	0.47	N/A	N/A
Equisolid-angle	1.22	0.23	N/A	N/A
Orthogonal	1.67	0.37	N/A	N/A
Stereographic	0.35	0.30	N/A	N/A

**Table 12:** RMS-residuals of lens distortion (pixel).

Projection Model	Test Object Type			
	Plane	V	A	Room
Perspective	0.65	1.89	0.24	0.26
Equidistant	0.75	4.22	N/A	N/A
Equisolid-angle	0.90	9.91	N/A	N/A
Orthogonal	0.72	0.73	N/A	N/A
Stereographic	0.29	4.47	N/A	N/A

**Table 13:** Coverage ratio of image points (mean, %).

Projection Model	Test Object Type			
	Plane	V	A	Room
Perspective	85	63	93	91
Equidistant	73	39	88	95
Equisolid-angle	70	40	86	93
Orthogonal	81	56	90	93
Stereographic	80	44	90	96

**Table 14:** RMS-residuals of IOPs (pixel).

Projection Model	Test Object Type			
	Plane	V	A	Room
Perspective	0.87	1.99	0.34	0.39
Equidistant	0.48	4.28	N/A	N/A
Equisolid-angle	0.44	10.15	N/A	N/A
Orthogonal	0.40	0.75	N/A	N/A
Stereographic	0.35	4.55	N/A	N/A

**Table 15:** Explanation of image groups shown in Figure 8.

Group	Location Number	κ (°)	Purpose
A	1, 2, 3	0	To reduce correlation of $x_{p}$-X0, $y_{p}$-Y0, and f-Z0
	4, 5, 6	90	
B	7, 8	0	To reduce correlation of $x_{p}$-X0, and $y_{p}$-Y0, and to increase image coverage
	9, 10	90	
C	11, 12	0	
	13, 14	90	

**Table 16:** Image-set configuration for additional experiments.

Image-Set	Included Image Group	Number of Images
1	A, B	10
2	A, C	10
3	A, B, C (all image groups)	14

**Table 17:** Correlation between the orientation parameters (image-set 1).

Projection Model	IOP	EOP					
		X0	Y0	Z0	ω	φ	κ
Equidistant	$x_{p}$	0.14	0.08	0.07	0.35	0.49	0.01
	$y_{p}$	0.12	0.17	0.01	0.41	0.32	0.08
	f	0.07	0.01	0.71	0.01	0.01	0.01
Equisolid-angle	$x_{p}$	0.13	0.08	0.06	0.35	0.49	0.00
	$y_{p}$	0.11	0.17	0.01	0.41	0.32	0.07
	f	0.08	0.01	0.71	0.01	0.01	0.01
Orthogonal	$x_{p}$	0.13	0.07	0.05	0.34	0.47	0.00
	$y_{p}$	0.10	0.15	0.01	0.38	0.31	0.05
	f	0.09	0.01	0.72	0.01	0.01	0.01
Stereographic	$x_{p}$	0.16	0.07	0.08	0.35	0.49	0.01
	$y_{p}$	0.14	0.18	0.02	0.42	0.33	0.09
	f	0.07	0.01	0.70	0.01	0.01	0.01

**Table 18:** Correlation between the orientation parameters (image-set 2).

Projection Model	IOP	EOP					
		X0	Y0	Z0	ω	φ	κ
Equidistant	$x_{p}$	0.09	0.12	0.11	0.38	0.53	0.10
	$y_{p}$	0.09	0.13	0.09	0.51	0.36	0.23
	f	0.14	0.01	0.47	0.03	0.02	0.03
Equisolid-angle	$x_{p}$	0.09	0.12	0.11	0.46	0.45	0.17
	$y_{p}$	0.09	0.13	0.10	0.44	0.43	0.16
	f	0.15	0.01	0.46	0.03	0.02	0.03
Orthogonal	$x_{p}$	0.12	0.10	0.08	0.41	0.36	0.14
	$y_{p}$	0.12	0.10	0.08	0.36	0.41	0.08
	f	0.22	0.01	0.46	0.02	0.02	0.02
Stereographic	$x_{p}$	0.09	0.11	0.09	0.52	0.40	0.23
	$y_{p}$	0.09	0.12	0.07	0.39	0.51	0.11
	f	0.16	0.01	0.47	0.01	0.03	0.01

**Table 19:** Correlation between the orientation parameters (image-set 3).

Projection Model	IOP	EOP					
		X0	Y0	Z0	ω	φ	κ
Equidistant	$x_{p}$	0.14	0.09	0.15	0.34	0.52	0.11
	$y_{p}$	0.08	0.17	0.06	0.43	0.31	0.20
	f	0.19	0.00	0.45	0.02	0.03	0.02
Equisolid-angle	$x_{p}$	0.14	0.09	0.15	0.40	0.46	0.16
	$y_{p}$	0.08	0.17	0.07	0.39	0.36	0.15
	f	0.18	0.00	0.45	0.02	0.02	0.02
Orthogonal	$x_{p}$	0.16	0.08	0.13	0.34	0.37	0.12
	$y_{p}$	0.10	0.15	0.07	0.33	0.31	0.11
	f	0.20	0.00	0.44	0.02	0.03	0.02
Stereographic	$x_{p}$	0.15	0.08	0.13	0.36	0.51	0.13
	$y_{p}$	0.09	0.16	0.05	0.43	0.32	0.21
	f	0.15	0.00	0.45	0.02	0.04	0.02

**Table 20:** Absolute error of principal point coordinates (pixel).

Projection Model	Image-Set					
	1		2		3	
	$x_{p}$	$y_{p}$	$x_{p}$	$y_{p}$	$x_{p}$	$y_{p}$
Equidistant	0.27	0.30	0.77	0.13	0.71	0.02
Equisolid-angle	0.56	0.06	0.28	0.15	0.44	0.14
Orthogonal	0.25	0.08	0.09	0.40	0.04	0.10
Stereographic	0.39	0.12	0.29	0.23	0.01	0.18

**Table 21:** Absolute error of focal length (pixel).

Projection Model	Image-Set		
	1	2	3
Equidistant	1.97	0.04	0.04
Equisolid-angle	1.25	0.22	0.46
Orthogonal	1.91	0.58	0.16
Stereographic	0.61	0.96	0.70

**Table 22:** RMS-residuals of lens distortion (pixel).

Projection Model	Image-Set		
	1	2	3
Equidistant	7.48	0.18	0.15
Equisolid-angle	2.47	0.34	0.39
Orthogonal	1.21	0.32	0.08
Stereographic	3.12	1.50	0.75

**Table 23:** Coverage ratio of image points (%).

Projection Model	Image-Set		
	1	2	3
Equidistant	49	85	85
Equisolid-angle	49	86	86
Orthogonal	69	90	90
Stereographic	58	77	77

**Table 24:** RMS-residuals of IOPs (pixel).

Projection Model	Image-Set		
	1	2	3
Equidistant	6.12	0.85	0.70
Equisolid-angle	1.63	0.28	0.39
Orthogonal	0.38	0.35	0.11
Stereographic	2.45	0.78	0.61
